# Cardiac surgery, a right target for hyperoxia?

**DOI:** 10.1186/s13054-016-1347-9

**Published:** 2016-06-16

**Authors:** Julie Boisramé-Helms, Peter Radermacher, Pierre Asfar

**Affiliations:** Service de Réanimation Médicale, Nouvel Hôpital Civil, Hôpitaux Universitaires de Strasbourg, Strasbourg, France; EA 7293, Fédération de Médecine Translationnelle de Strasbourg (FMTS), Faculté de médecine, Université de Strasbourg, Strasbourg, France; Institut für Anästhesiologische Pathophysiologie und Verfahrensentwicklung, Universitätsklinikum Ulm, Helmholtzstrasse 8/1, 89081 Ulm, Germany; Département de Réanimation Médicale et de Médecine Hyperbare Centre Hospitalier Universitaire, Angers, 49933 Cedex 9 France; Laboratoire de Biologie Neurovasculaire et Mitochondriale Intégrée, CNRS UMR 6214—INSERM U1083, Université Angers, PRES L’UNAM, Angers, France

## Abstract

In perioperative cardiac surgery period, supra-physiological arterial oxygen partial pressures is common practice, although there is no clear evidence of any benefit. Smit et al. have shown that a “conservative” approach did not improve hemodynamics, decrease oxidative stress or myocardial tissue damage, but was not associated with major deleterious event either. Here, we outline major oxygen friend or foes properties, which may partly explain the study results, and place the clinical trial from Smit et al. in a global context.

## Introduction

Targeting supra-physiological arterial oxygen partial pressures (PaO_2_) during the perioperative period of cardiac surgery is common practice [1], but why? The surgical trauma per se and the ischemia–reperfusion sequence associated with cardiopulmonary bypass (CPB) cause hyper-inflammation and excessive release of reactive oxygen species (ROS). Any alveolar, arterial, and/or tissue hypoxia also triggers hyper-inflammation, and cardiac surgery may induce an imbalance between tissue oxygen delivery (DO_2_) and oxygen consumption (VO_2_) due to myocardial dysfunction, vasoplegia, microcirculation alterations, hypothermia, anemia, and hypovolemia. High inspiratory oxygen concentrations (FiO_2_) can theoretically counteract this problem, but so far the optimal targets for PaO_2_ during CPB and/or the immediate postoperative ICU stay remain open [1].

## Main text

Recently, Smit et al. [2] investigated whether a “conservative” oxygen approach targeting a “near-physiological” PaO_2_ of 130–150 and 80–100 mmHg during CPB and in the first 12 hours of ICU stay, respectively, would improve hemodynamics, reduce oxidative stress, and attenuate myocardial damage and visceral organ dysfunction. The control group received standard care; that is, PaO_2_ targets were 200–220 and 130–150 mmHg during CPB and early ICU stay, respectively. The main results were that the “conservative” approach did not improve hemodynamics, and neither attenuated oxidative stress (assessed by the plasma isoprostane levels and the ex-vivo ROS release in polymorphonuclear leukocytes) nor myocardial tissue damage (assessed by troponin-T levels and myocardial creatinine kinase (CK-MB) activity). However, the “conservative” strategy was not associated either with major hyperlactatemia, visceral organ dysfunction (assessed by plasma creatinine levels), or hypoxic events (defined by PaO_2_ < 55 mmHg).

Hyperoxia has “friend-and-foe” properties, both in critically ill patients in general [3] and specifically in cardiac surgery patients [1]. Perioperative hyperoxia per se can improve oxidative killing of bacteria [3], since bactericidal properties of neutrophils depend on the PO_2_ in the contaminated tissue. In fact, the most recent meta-analysis on this subject including nine studies comprising 5103 patients concluded that perioperative hyperoxia may reduce surgical site infection, in particular in patients undergoing colorectal surgery [4]. Nevertheless, given the negative result of the large-scale, multicenter, randomized “Supplemental Oxygen and Complications After Abdominal Surgery” (PROXI) trial, the most recent Cochrane review [5] concluded that, so far, robust evidence is lacking to recommend the use of high FiO_2_ during the perioperative period, and may even be responsible for adverse events. In fact, the use of perioperative hyperoxia has been cautioned by the data on the long-term follow-up (up to 3 years) of the PROXI trial: hyperoxia increased long-term mortality, and this was due to the subgroup of patients undergoing cancer surgery [6], who showed a reduction in cancer-free survival [7].

Perioperative hyperoxia might also allow for myocardial ischemia-preconditioning in cardiac surgery [8]. Conversely, it may exacerbate the transitory CPB-related pulmonary injury due to increased oxidative stress and aggravated systemic inflammatory response [9]. Finally, hyperoxia may also affect hemodynamics during and after cardiac surgery due to systemic vasoconstriction, decreased heart rate, and direct negative inotropic effects, overall decreasing cardiac output [2, 3]. It is well established that hyperoxia-induced vasoconstriction is particularly pronounced in the coronary circulation [10], thus possibly increasing susceptibility to myocardial ischemia and ischemia–reperfusion-induced injury in patients with coronary artery disease (CAD) [2, 3]. In nonhypoxemic patients, the AVOID trial [11] showed that high-flow O_2_ therapy during ST-segment elevation myocardial infarction increased CK-MB activity, which coincided with higher Troponin-I levels (*p* = 0.12) and infarct size (*p* = 0.06). Interestingly, albeit not significantly different (*p* = 0.11), mortality at hospital discharge was 2.5-fold higher in the normoxia group. Hyperoxia pre*-*treatment (as during cardiac surgery) may prolong the “window of opportunity” prior to myocardial ischemia: high-flow O_2_ increased the time to onset of both pacing-induced and exercise-induced myocardial ischemia in CAD patients [12, 13].

What do we learn from the study by Smit et al. [2]? The “conservative” approach did not beneficially affect the parameters assessed, but had no deleterious side effects either. Signs of possible tissue hypoperfusion (i.e., lactate levels) only showed a mild increase (maximum 2 mmol/l) independently of the group assignment, and none of the patients showed major organ dysfunction. It remains unclear whether the “conservative” approach substantially altered DO_2_ and/or VO_2_: the cardiac index and total hemoglobin content were comparable, and the time-weighted PaO_2_ values (220 vs*.* 157 mmHg, 214 vs*.* 147 mmHg, and 107 vs*.* 90 mmHg during CPB, aortic-cross clamping, and ICU stay, respectively) suggest that there were no major differences in hemoglobin O_2_ saturation. Since neither glycemia nor data on insulin requirements are reported, it remains open whether there was any difference in metabolic substrate utilization: increasing FiO_2_ was reported to reduce O_2_ uptake in critically ill patients and, eventually, switch energy metabolism to preferential carbohydrate use [3]. Finally, as the authors acknowledge themselves, the systematic dexamethasone administration after induction of anesthesia may have mitigated any further effect of the different O_2_ strategy. Nevertheless, the results presented by Smit et al. are of major interest and clinical importance given the current practice comprising administration of high FiO_2_ levels and targeting supra-physiological PaO_2_ values in cardiac surgery. Previous studies have already suggested that more conservative O_2_ targets might be beneficial [14, 15]. However, in these studies the “hyperoxic” groups had PaO_2_ levels of 400–500 mmHg during CPB, and the “lower”, so-called “normoxic” groups were titrated to PaO_2_ levels of 140 mmHg [14] and 200–250 mmHg [15], respectively; that is, close to the targets of the hyperoxic “control” arm in the present investigation. Moreover, PaO_2_ strategies were only modified during CPB, whereas Smit et al. also targeted the early ICU stay.

## Conclusion

Despite some study limitations, Smit et al. have to be commended for performing an elegant clinical trial on routine procedures that had so far never undergone rigorous testing under randomized, controlled conditions. The results of their much larger “Optimal Oxygenation in the Intensive Care Unit (O2-ICU)” trial (ClinicalTrials.gov NCT02321072) comparing high-normal vs*.* low-normal (120 vs*.* 75 mmHg) PaO_2_ targets in ICU patients is therefore eagerly awaited.

## Abbreviations

CAD, coronary artery disease; CPB, cardiopulmonary bypass; CK-MB, myocardial creatinine kinase; DO_2_, oxygen delivery; FiO_2_, inspiratory oxygen concentrations; PaO_2_, arterial oxygen partial pressures; ROS, reactive oxygen species; VO_2_, oxygen consumption
